# THE EFFECT OF SUBJECTIVE AND OBJECTIVE RELATIONSHIPS ON THE LIFE SATISFACTION AND HEALTH OF OLDER ADULTS

**DOI:** 10.1093/geroni/igac059.2642

**Published:** 2022-12-20

**Authors:** Meeryoung Kim

**Affiliations:** Daegu University, Daegu, Kyongsang-bukto, Republic of Korea

## Abstract

This study examines the effects of subjective and objective relationships among older adults on life satisfaction. Human beings are social animals and must live in relationships with people. In particular, in a situation where social networks decrease in old age, objective or subjective social relationships that can receive help when needed, and satisfaction with such relationships can improve the quality of life in old age. This study used the sixth additional wave (2016) and the seventh wave (2017) of the Korean Retirement Income Study. The subjects of this study were older adults who are aged 65 and older, and the sample size was 3,423. The number of objective relationships, subjective social support, relational satisfaction, and having a spouse and children were used as independent variables. Life satisfaction and health were used as dependent variables. Demographic variables were controlled. Multiple regression was used for data analysis. The more objective relationships there were, the higher the life satisfaction and health. Subjective social support also significantly increased life satisfaction and health. Satisfaction with interpersonal relationships also significantly increased both life satisfaction and health. Having a spouse made a significant difference in affecting life satisfaction and health. The results showed that both subjective and objective relationships in old age improved the quality of life of older adults, but the presence or absence of children made no significant difference in affecting life satisfaction or health. This implies that as you get older, you may find that having a spouse becomes very important.

